# Harnessing the Potential of Human Pluripotent Stem Cells and Gene Editing for the Treatment of Retinal Degeneration

**DOI:** 10.1007/s40778-017-0078-4

**Published:** 2017-04-18

**Authors:** Patrick Ovando-Roche, Anastasios Georgiadis, Alexander J. Smith, Rachael A. Pearson, Robin R. Ali

**Affiliations:** Department of Genetics, UCL Institute of Ophthalmology, 11-43 Bath Street, London, EC1V 9EL UK

**Keywords:** Vision impairment, Retina, Photoreceptors, Induced pluripotent stem cells, Disease modelling, Gene editing

## Abstract

**Purpose of Review:**

A major cause of visual disorders is dysfunction and/or loss of the light-sensitive cells of the retina, the photoreceptors. To develop better treatments for patients, we need to understand how inherited retinal disease mutations result in the dysfunction of photoreceptors. New advances in the field of stem cell and gene editing research offer novel ways to model retinal dystrophies in vitro and present opportunities to translate basic biological insights into therapies.

This brief review will discuss some of the issues that should be taken into account when carrying out disease modelling and gene editing of retinal cells. We will discuss (i) the use of human induced pluripotent stem cells (iPSCs) for disease modelling and cell therapy; (ii) the importance of using isogenic iPSC lines as controls; (iii) CRISPR/Cas9 gene editing of iPSCs; and (iv) in vivo gene editing using AAV vectors.

**Recent Findings:**

Ground-breaking advances in differentiation of iPSCs into retinal organoids and methods to derive mature light sensitive photoreceptors from iPSCs. Furthermore, single AAV systems for in vivo gene editing have been developed which makes retinal in vivo gene editing therapy a real prospect.

**Summary:**

Genome editing is becoming a valuable tool for disease modelling and in vivo gene editing in the retina.

## Introduction

### Inherited Retinal Degenerative Diseases

Vision impairment is a global health issue estimated to affect more than 285 million people worldwide [1]. Over 50% of all visual impairment cases in the developing world are the result of dysfunction and/or loss of photoreceptors, a specialized type of neuron that performs the essential first step in transforming light into vision [2]. Inherited retinal diseases such as retinitis pigmentosa (RP), Leber congenital amaurosis (LCA), Stargardt disease, as well as more complex and heterogeneous retinal diseases such as age-related macular degeneration (AMD), are among the most common types of retinal degeneration [3]. Inherited retinal diseases have been associated with mutations in more than 200 different genes (see http://www.sph.uth.tmc.edu/Retnet) [4, 5]. As a result, the onset of hereditary disease and the speed of progression can be highly variable. This contrasts with age-related macular degeneration (AMD) which specifically affects older adults and where dysfunction of photoreceptors is thought to be caused by cellular senescence of retinal pigment epithelium cells (RPE), a photoreceptor support cell [6–8].

**Fig. 1:**
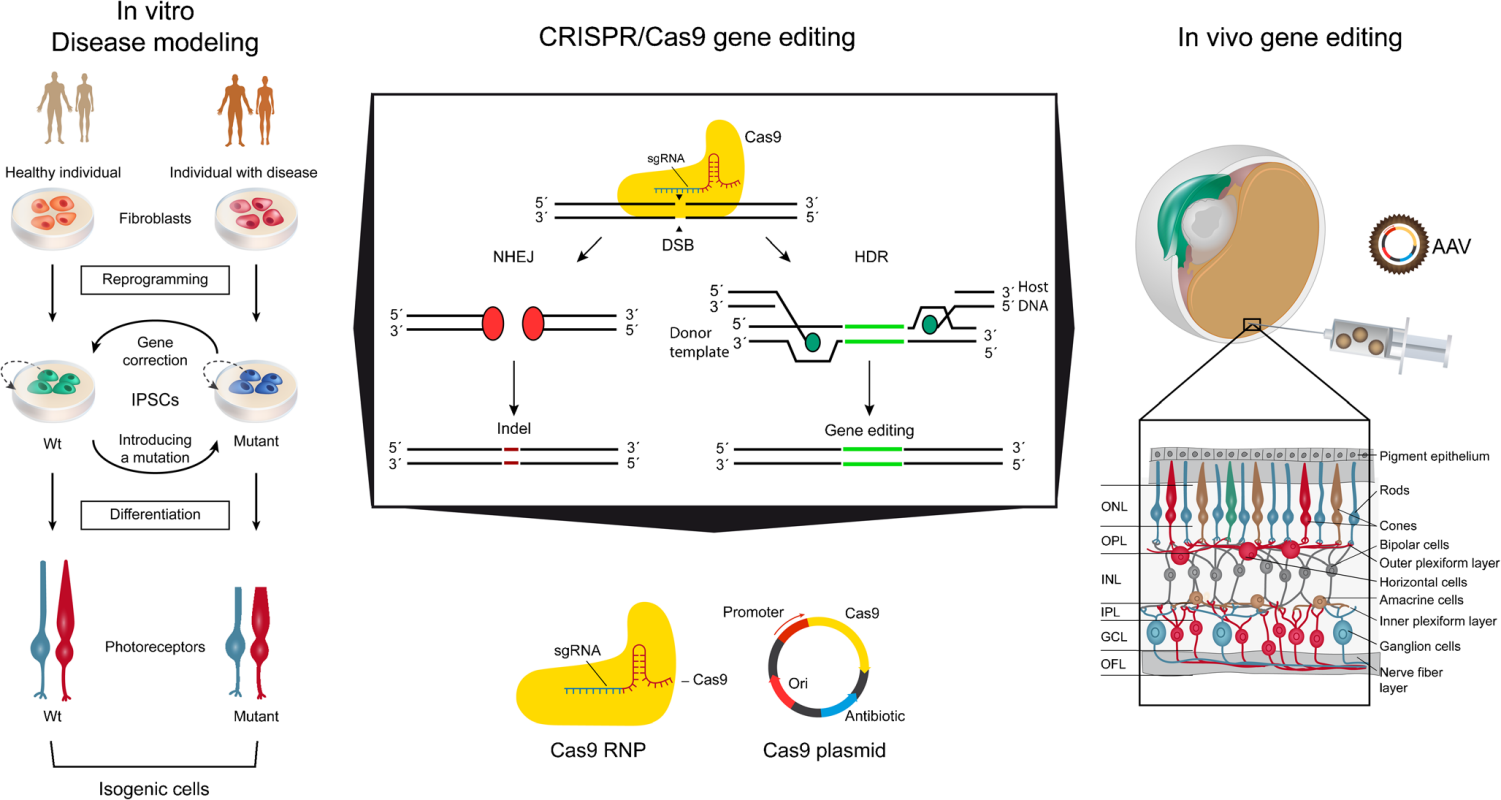
Schematic diagram depicting how CRISPR/Cas9 gene editing can be harnessed for in vitro disease modelling and in vivo gene editing. *CRISPR/Cas9 gene editing panel:* Cas9 nuclease in complex with a gRNA can generate specific double-stranded breaks (DSB) in the host DNA. Cell’s DNA repair mechanism will repair the DSB by either error-prone NHEJ or error-free HDR. NHEJ-mediated gene editing will most likely result in the introduction of insertions and deletions (indel, *red lines*) that will lead to premature STOP codon formation. HDR-mediated gene editing, in the presence of a homologous DNA template (*green lines*), will introduce precise genomic changes in the host’s DNA. Most popular CRISPR/Cas9 gene editing approaches are an RNP approach, where Cas9 protein is complexed with gRNA for delivery, and a plasmid approach, where Cas9 cDNA, gRNAs and a reporter are usually overexpressed from one, two or more vectors. *In vitro disease modelling panel:* Shows how fibroblasts can be sampled from healthy and affected individuals and reprogrammed into human iPSCs. CRISPR/Cas9 gene editing can then be used to introduce disease-causing mutations of interest in healthy iPSCs or correct them in affected iPSCs to generate isogenic iPS cell line pairs. These can then be differentiated to photoreceptors for disease modelling purposes. *In vivo gene editing:* cDNA containing Cas9 gene editing tool can be packed into AAV for subretinal injection to target different populations of retinal cells. The retina is a layered structure composed of three layers of cells connected by two synaptic layers: the inner plexiform layer (IPL) and the outer plexiform layer (OPL). At the outer most region, cone and rod photoreceptor cells form the outer nuclear layer (ONL). The inner nuclear layer (INL) is composed of bipolar, amacrine, horizontal and Müller glia cells. Lastly, the inner most layer, the ganglion cell layer (GCL) is comprised of retinal ganglion cells and displaced amacrine cells. The optic fiber layer (OFL) contains retinal nerve fibers that exit the eye through the optic nerve

Compared with many other parts of the nervous system, the eye represents a highly accessible and (at least partially) immune-privileged system [9–11]. It is therefore unsurprising that it has become a focus of significant translational research efforts. Common to all vertebrate retinas is a highly stratified structure, composed of three layers of cells connected by two synaptic layers. The outer retina is composed of photosensitive cone and rod photoreceptor cells, which form the outer nuclear layer (ONL), connecting to interneurons including bipolar, amacrine and horizontal cells in the inner nuclear layer (INL) (Fig. 1). Notably, most forms of inherited retinal diseases affect photoreceptors or their support cells (e.g. RPE), resulting in photoreceptor death, but the inner retina (i.e. bipolar, amacrine, horizontal and ganglion cells) remains largely unaffected [3, 12, 13]. This makes the retina an attractive recipient for novel therapeutic approaches including gene and cell therapy and the use of implant devices.

### Gene and Cell Therapies

Archetypal gene therapy aims to provide a normal copy of the gene that is faulty by delivering it via a viral vector [14]. The most effective vectors for retinal transduction are those based on adeno-associated viruses (AAV). These vectors can mediate efficient long-term gene transfer to both photoreceptors and RPE. Recombinant AAV vectors can be constructed with a number of different capsids (i.e. serotypes) that can lead to transduction of different retinal cell types depending on route of administration. AAVs are relatively small viruses with a single-stranded DNA genome. They are non-pathogenic to humans, and depending on their method of delivery, they can penetrate deep into tissues providing very good levels of cellular transduction. In the retina, subretinal delivery is currently the route of administration of choice with serotypes such as AAV5 and AAV8 providing highly efficient photoreceptor transduction. While the capacity of AAV vectors is relatively small when compared to other viral vectors, with a maximum capacity of approximately 5 kb of insert DNA, it is large enough to accommodate most genes. In preclinical studies over the last 15 years, the utility of AAV vectors for gene supplementation for recessively inherited degenerations has been demonstrated in a number of animal models leading to long-term phenotypic rescue [15, 16].

The success in preclinical development has led to a number of phase I/II clinical trials to treat inherited retinal degenerative diseases using gene therapy [17–23]. In the first trials, patients carrying a defective *RPE65* gene, which causes a form of LCA, received a subretinal injection of an AAV vector carrying a human RPE65 complementary DNA (cDNA) under the control of either a human RPE65 promoter [17] or a ubiquitously expressed actin promoter [20, 23]. In each case, subretinal delivery of an AAV vector in patients was shown to be safe. Additional findings included improvement of visual function, although these improvements varied between patients and were modest overall. More recent reports have further demonstrated efficacy but have also underlined the importance of achieving the correct levels of gene expression for robust and sustained rescue [24–26]. Despite the success of these trials, a gene supplementation approach has its limitations; in particular, it requires the presence of the affected cells and is most effective in the early stages of disease progression. Another limitation is the length of the cDNA that the AAV can package for delivery [27–30], which prevents replacement of very large genes, such as ATP-binding cassette subfamily a member 4 (*ABCA4*, ∼7.3 Kb), which causes Stargardt disease [31, 32] and centrosomal protein of 290 kDa (*CEP290*, ∼7.9 kb) which causes another form of LCA [33]. Moreover, it can only be used for single-gene defects involving a known gene.

### Stem Cell-Derived Photoreceptors

Cell replacement therapy offers a therapeutic approach for patients with advanced disease where there has been extensive photoreceptor loss. It may also permit treatment of conditions with non-genetic or unknown cause. We, and others, have carried out a variety of studies in the area of photoreceptor derivation and transplantation. Ground-breaking work on murine embryonic stem cells (ESCs) by Sasai and colleagues [34•] showed that ESCs cultured in 3D suspension in the presence of matrices, such as Matrigel, spontaneously form self-organising embryoid bodies that support the morphological differentiation of retinal cells. This so-called 3D culture technique has since been shown to sustain the differentiation of murine photoreceptors up to equivalents of the first postnatal week [35]. We, and others, have shown that donor photoreceptor cells from early mouse postnatal retina as well as ESC-derived mouse photoreceptor precursors can be transplanted into the degenerative retinas of adult mice [35–43]. Following transplantation, if present in sufficient numbers, these cells are capable of restoring aspects of visual function [42, 44–47]. Several laboratories have also described the derivation of photoreceptors from human ESCs and iPSCs [48–54, 55•, 56••]. Canto-Soler and colleagues were the first to show human pluripotent stem cells (hPSCs)-derived retinal organoid containing photoreceptors that bear key photoreceptors structures and are responsive to light in vitro [56••]. Their protocol uses a 3D to 2D to 3D multi-stage cell culture differentiation process that takes advantage of the ability of hPSCs to spontaneously differentiate towards the ectoderm pathway under neural induction culture conditions. In this protocol, unlike previous neuro-differentiation approaches [57–59], typically costly SMAD inhibitors are avoided in the neural induction process. Instead, hPSCs are allowed to form embryoid bodies (EBs) in suspension, then allowed to grow in a monolayer until “horseshoe”-shaped neural retinal-like structures appear spontaneously. These can then be dissected manually and cultured in suspension for up to 27 weeks to form mature retinal organoids. This hPSCS retinal differentiation protocol has an initial 3D to 2D step as others have previously described [48, 52, 53]. Among the advantages of these protocols is the ability to control the starting number of cells and, therefore, reduced initial variability. However, issues may arise when the resulting EBs are then grown on a 2D monolayer to further develop, as the presumptive neural retina structures are dissected manually. This appears to be a key stage in variability between different laboratories using the same protocol, as the criteria used to define the presumptive neural retinal structures is subjective. Canto-Soler and colleagues found that photoreceptors start to express mature markers, such as Rhodopsin, S-Opsin and LM-opsin, by week 17 and that these photoreceptors demonstrate light sensitivity by week 27. Goureau and colleagues on the other hand, used a simpler 2D to 3D cell culture system, where human iPSCs cells are allowed to become confluent on a monolayer, prior to neural induction [55•]. They take advantage of the cells’ ability to self-form into neural retinal-like structures even within the 2D cell culture conditions; these could then be dissected and further differentiated in 3D suspension, going on to correctly express many markers of retinal commitment and even opsin expression. While this protocol demonstrates a simpler way to differentiate iPSCs to neural retina, it failed to support the morphological differentiation of photoreceptors, including the formation of outer segments (OS), as described by Canto-Soler and colleagues [56••]. However, while Canto-Soler’s protocol supported Opsin expression and light sensitivity of hPSC-derived photoreceptors, the transmission electron microscopy (TEM) data showed little evidence of mature OS formation. Recently, we have adapted some of the protocols described previously and have found that it is possible to generate short OS-like structures from hESC-derived retinal organoids (Gonzalez-Cordero et al., in preparation). A recent report by Takahashi and colleagues showed that transplantation of immature hPSC-derived photoreceptors into the subretinal space of nude rats allows for the subsequent formation and maturation of OS structures [60••]. This suggests that the hPSC-derived photoreceptors described to date may all have the capacity to form OS but lack sufficient instruction or support from the in vitro environment to be able to do so.

A major issue currently limiting the widespread utility of hPSC-derived cells is the variability in efficiency between protocols and even the reproducibility of the same protocol by different laboratories. There are many potential variables that may be introduced, including the cell line used and its passage number, the different batch numbers of commercially produced reagents used and the subjective choice of neural retina resembling structures. Robust methods of quantifying the purity and viability, as well as the developmental stage, of photoreceptors will be important. As a step towards this, we, together with Sowden and colleagues, have used cluster of differentiation (CD) cell surface markers to quantify and purify stage-specific photoreceptors in mouse ES-derived photoreceptors and mouse and human retinal samples [40, 61]. Among these, CD73 allows for the enrichment of rod precursor cells [38, 40, 61]. Furthermore, pilot experiments using human retinal samples indicated that a similar CD panel could be used to assess and quantify the maturation stage of hPSC-derived photoreceptors, especially since human iPSC-derived photoreceptors and adult human retina are reported to express CD73 [55•, 61]. This approach, if successful in conjunction with human retinal organoids, may be useful for cell therapy purposes, as it circumvents the use of reporter markers in transgenic hPSC lines that cannot be used clinically. A CD panel could also be a good way to characterise and quantify the extent of retinal differentiation in individual batches, since previous studies have demonstrated that different hPSCs differentiate along the retinal lineage with varying efficiencies and with the resulting retinal cells almost always co-existing alongside non-retinal cells [48, 52, 54, 62–64]. An additional point to note is the need to develop robust criteria for defining what constitutes commitment of the stem cell to differentiate into a given cell type. At present, the field relies heavily on the presence/absence of various photoreceptor-specific proteins, typically assessed by immunocytochemistry. We have already shown that such methods of assessment can lead to erroneous conclusions about how well a cell has adopted a given fate [43] and therefore it will be important to assess the derived cells with greater thoroughness, using other assays such as RNAseq, morphology and functionality.

A challenge when differentiating human ES or iPSCs to retinal neurons using 3D culture methods is that, like mouse ES retinal differentiation, human ESC/iPSC retinal differentiation follows a time course similar to that of normal development, which for the human organoids is many months (Gonzalez-Cordero et al., in preparation); this leads to a large proportion of differentiated organoids becoming necrotic. This is a major hurdle, since photoreceptor OSs form late in development and need long periods of cell culture to form (Gonzalez-Cordero et al., in preparation). Recent findings from related ESC disciplines suggests that upgrading from the classic cell culture plate differentiation approach to the use of bioreactors can improve differentiation and viability, providing cells with improved aeration and distribution of nutrients, as well as apparently encouraging formation of 3D structures [65–68]. Preliminary work in our group suggests that bioreactor technology may represent a stepping stone to upscaling the cell culture process for therapeutic applications, together with the ability to improve the survival and abundance of mature photoreceptors (Ovando-Roche et al., in preparation).

Regardless of these challenges, however, it is clearly possible to generate functional photoreceptors from hPSCs. This raises the question of how best to use these advances to model retinal disease in vitro.

## Using Human Pluripotent Stem Cells as Retinal Disease Modelling Tools

The inability of animal models to reflect human disorders accurately may have contributed to disappointing outcomes in many clinical trials of drugs and therapies. The revolution in somatic cell reprogramming that has enabled the generation of human iPSCs from adult tissue [69], coupled with recent advances in genome-editing technology [70–72], now allows us to investigate cellular aspects of human neurodegenerative disease in vitro. This not only potentially circumvents the dependence on scarce primary patient tissue and animal models to develop a therapy but may also facilitate a patient-tailored approach. We can now derive human iPSCs from patients and differentiate these into various tissues to model some aspects of disease in vitro [73•, 74]. Not only do these cells offer the opportunity to model cellular phenotypes but also provide a platform for high throughput drug screening and cell transplantation studies.

While it is not possible to model all aspects of a condition using iPSC-based disease modelling, it can be a very powerful tool for investigating cellular pathology. A major limitation, however, is the differentiation and phenotypic variability that is observed even in human iPSCs derived from the same donor [75, 76]. This is exacerbated further when comparing between unrelated hPSC lines. Even if the cellular phenotype of a given mutation is strong and highly penetrant, it may be lost due to genetic and epigenetic background differences [75–79]. A powerful approach to overcome this hurdle is to use gene editing tools that enable precise editing of endogenous hPSC genomic sequences [80]. In this scenario, gene editing can be used to correct the disease-causing mutation in a patient-derived iPSC line to generate a pair of isogenic iPSC lines that differ only in the disease-causing mutation, providing a less variable and more faithful disease modelling system. Alternatively, the same technology could be used, for example, to insert disease-causing monogenic mutations in unaffected hPSCs to generate isogenic pairs for disease modelling. The former option has the advantage that for recessive diseases only one allele needs to be repaired, whereas the latter strategy would need both alleles to be mutated. However, the latter approach would have the added advantage of reducing costs and research time, as obtaining patient samples and generating iPSCs is both costly and time consuming. These gene editing strategies will prove invaluable for studying human biology and disease.

In one of the first human iPSCs studies to model retinal disorders, Gamm and colleagues focused on Best disease, an inherited retinal dystrophy of the macula that leads to progressive and irreversible central vision loss [81]. In this disease, mutations in the RPE gene bestrophin 1 (*BEST1*) result in an accumulation of waste products from shed photoreceptor OS in the RPE cells, resulting in RPE dysfunction and secondary photoreceptor death. In this study, human iPSCs were derived from both patients and their unaffected siblings and differentiated into functional RPE cells. Affected iPS cell-derived RPE cells displayed delayed rhodopsin protein degradation after photoreceptor OS phagocytosis, compared to unaffected iPS cell-derived RPE. These findings indicated, for the first time in a human iPS cell-derived RPE cell, that *BEST1* mutations cause defective photoreceptor OS handling [81]. In another iPS disease modelling study, Stone and colleagues modelled disease resulting from usherin (*USH2A*) mutations, which lead to autosomal recessive RP [82]. They derived iPSCs from a patient with a *USH2A* mutation and differentiated the resulting cells towards a photoreceptor fate, comparing their results to unrelated, unaffected iPSC-derived photoreceptors. The patient-derived *USH2A* mutant photoreceptors had upregulation of GRP78 and GRP94, suggesting that mutations in *USH2A* can cause endoplasmic reticulum stress, which occurs frequently in neurodegenerative diseases [82]. In a study by Cheetham and colleagues, iPSCs derived from a healthy individual and a patient bearing an LCA-causing mutation in *CEP290* were differentiated into fibroblasts, RPE and photoreceptors [83]. They found that CEP290-LCA-derived fibroblasts and photoreceptors showed a reduction in the number of ciliated cells, as well as cilia length, in line with the fact that CEP290 has been shown to be important in regulating cilia assembly and development [84, 85]. The phenotype could be rescued by treating the CEP290-LCA cells with antisense morpholino oligonucleotides, which blocked the aberrant splicing of *CEP290* [83].

The first reported studies demonstrate the potential of iPSC retinal disease modelling. However, there are some key issues to consider when interpreting the results and setting up future studies. Robust methods of quantification and, ideally, a comparison of several cell lines should be used; this is of even greater importance when isogenic controls are not available. The high variability in differentiation capacities between different ESCs and/or iPSCs is well documented. Even when retinal organoids are derived from the same iPSC line at the same time and are cultured under the same conditions, they can reach maturity at different times. In addition, some organoids may fail to differentiate completely, while others differentiate successfully. Such differences may arise because each organoid is likely to have different cell type content and cell-to-cell interactions. In light of such variations, comparing subtle phenotypic changes may be problematic. Repeated batch differentiation, coupled with robust quantification approaches, such as western blotting, qRT-PCR and/or RNAseq and FACS analysis will be key to obtaining meaningful and consistent results. These approaches will allow analysis of specific cell types instead of whole organoid analysis, where variation in cellular composition may create or obscure a phenotype. Variability can also be minimised by using optimal controls. For example, Gamm and colleagues used iPSCs derived from unaffected siblings as controls [81]. While the siblings may not have an identical genetic and epigenetic make-up, it is a more reliable comparison than comparing iPSC-derived retinal organoids from two unrelated individuals as used in the other studies described here [82, 83]. The optimal control, especially if the disease to be modelled is not highly penetrant, would be the same patient-derived iPSC line but gene edited to correct the disease-causing mutation (i.e. isogenic pairs). Unfortunately, the gene editing technology required to generate such isogenic controls with relative ease, CRISPR/Cas9 gene editing, has only been available for the past 4 years [70–72].

## Stem Cells and Gene Editing

Genome editing technologies have been in the field since the 1990s (reviewed in [80]). Site-specific nucleases including zinc fingers (ZFN), transcription activator-like effectors (TALENS) and Cas9 proteins allow the introduction of precise double-strand breaks and gene modifications within the genome. Once one of these nucleases generates a DNA cut, the cell’s DNA repair mechanism is activated to avoid cell death, repairing the DNA break either by (error-prone) non-homologous end joining (NHEJ) or by (error-free) homology-directed recombination (HDR) (Fig. 1) [86–92]. NHEJ-mediated gene editing typically leads to the introduction of insertions and deletions (indels) in the genome, resulting in frameshift mutations that will disrupt full-length protein production through generation of a stop codon or result in a gene knockout, depending where the nucleases have created the DNA cut. On the other hand, HDR-mediated gene editing, in the presence of a homologous DNA template, can be used to modify the target DNA area with high precision. In the disease modelling context, the power of HDR-mediated gene editing can be harnessed by transfecting cells with vectors encoding one of these nucleases together with an engineered homologous DNA template that contains the desired genetic change (Fig. 1). While ZFN and TALENS are highly customizable DNA-binding proteins and can be harnessed to drive sequence-specific DNA targeting [93–96], engineering these proteins to bind to specific DNA targets, as well as achieving their robust delivery for this purpose, can be laborious and technically challenging [97]. By contrast, Cas9 nucleases are guided by two short sequences of RNA, a specificity-determining guide RNA sequence, CRISPR RNA (crRNA), and a transactivating crRNA (tracrRNA), which serves as scaffold for the Cas9 protein to form a complex (i.e. a protein:RNA complex) that forms Watson-Crick base pairs with the complementary DNA target sequence, resulting in a site-specific double-strand break [71, 72, 98] (Fig. 1). Advances in the field of CRISPR/Cas9 have rapidly led to the establishment of engineered Cas9 plasmid-based systems, where a Cas9 can be combined with a single chimeric guide RNA (gRNA), a fusion of the tracrRNA:crRNA duplex, for genome editing in eukaryotic cells at any genomic locus of interest [99–101]. While the CRISPR/Cas9 system is easier to use than its previous counterparts (i.e. TALENS and ZFN), some key issues still remain with regard to HDR-mediated gene editing, these include Cas9 plasmid integration, off-targeting and gene editing efficiency. For example, choosing between a plasmid-based approach or a ribonucleoprotein (RNP) CRISPR/Cas9 approach, where Cas9 protein forms a complex with synthetic gRNA [102, 103], is a key decision as each has different advantages and disadvantages. In the case of precise HDR-mediated gene editing for human iPSC disease modelling, either approach could be used. Plasmid-based gene editing allows for reporter expression which, unlike an RNP approach, will allow enrichment for the population containing the Cas9 gene editing toolkit. The use of an all-in-one plasmid approach where the reporter (e.g. puromycin or enhanced green fluorescence protein, EGFP), Cas9, gRNAs and repair template (i.e. donor template for HDR-mediated repair) are present on a single vector [100, 104] is also advantageous, as it provides obvious delivery advantages over a two or three plasmid system, where a given target cell may not receive all necessary plasmids to carry out gene editing. However, it is important to take into consideration the reporter or selection marker of choice as up to 30% plasmid background integration has been reported to occur when using a drug resistance cassette [105]. In some studies, use of fluorescent reporters such as EGFP may be preferable as FACS sorting could be used to isolate the EGFP-positive cell population (i.e. transduced cells) and, upon repeated passaging, to isolate the EGFP-negative population (i.e. transduced and free of plasmid integration). The disadvantages of using a plasmid approach, however, include that the plasmid will take 6–12 h to express the Cas9 gene editing tool kit, and it will remain in most cells for over 72 h, which means the Cas9 will remain active during this time, making off-targeting more likely [102]. If CRISPR/Cas9 gene editing is used for clinical applications, detailed evaluation of undesirable off-target modifications will be essential. Since mismatches at the 5′ end of the gRNAs are tolerated, the use of the wild-type Cas9 can lead to unintended off-target effects [106–108]. To address this issue, investigators have engineered modified or “evolved” Cas9 proteins [109, 110]. Among these, Cas9 nickase (Cas9n) carries a catalytic amino acid substitution (D10A) in the conserved RuvC nuclease domain, which converts this enzyme into a nickase meaning that Cas9n can only cleave one strand of DNA [110]. With this enzyme, the two gRNAs that are adjacent on opposite strands of the target site, coupled with two Cas9n molecules, would reduce off-targeting in two ways. First, since single-strand breaks are quickly repaired by the error-free base excision repair mechanism, genome integrity is maintained [111]. Second, off-targeting is greatly reduced by the two gRNAs that the Cas9n system needs to generate a double-strand break, since the likelihood of the two different gRNAs being complementary to other regions of the genome and close enough to each other to drive Cas9n-based cleavage is very low. Using the Cas9n system, Zhang and colleagues found that off-targeting was reduced 50–1500-fold. An additional approach to reduce off-targeting is to shorten the length of the gRNAs; this may reduce Cas9 activity but it greatly reduces off-targets mutations [106, 108].

The Cas9n RNP approach is an alternative way to gene edit using the CRISPR/Cas9 system for in vitro disease modelling. This method circumvents the problem of integration in plasmid-based systems and significantly reduces off-targeting, since the Cas9 RNP complex is rapidly degraded in cells within 24 h when delivered directly [102, 103]. This means that DNA breaks would occur for a shorter period of time and since there is no plasmid in this approach, there is no risk of integration. Furthermore, unlike the plasmid approach, since the Cas9 RNP is an active nuclease complex, it starts to gene edit as soon as it is transfected into the cells. Cas9 RNP approaches have been shown to be highly efficient in introducing indels and HDR-mediated precise gene edits [102, 103], although to our knowledge an all-in-one plasmid vs RNP approach has not been directly compared for the same target gene using the same gRNAs.

The importance of generating isogenic iPSC line pairs has been discussed earlier. Jaenisch and colleagues showed that gene editing can be used to generate isogenic pairs (e.g. affected and repaired) from human iPSCs or ESCs, leading to two cell lines that have the same genetic and epigenetic background and differ only in the disease-causing mutation [112]. In this study, ZFNs were engineered to target the α-synuclein (*SNCA*) locus, a gene commonly mutated in Parkinson’s disease. Using different gene editing strategies, they introduced two common mutations of SNCA in unaffected ESCs or corrected the mutation in a patient-derived iPSC line [112]. Co-electroporation of the ZFN expression plasmids and donor constructs, followed by selection of the transfected cells, led to a genome editing efficiency of ∼0.9% (3 out of 336 screened clones where targeted at the correct locus). Furthermore, using a positive and negative selection approach, they obtained a maximum efficiency of ∼22% (9 out of 41 screened clones correct for the desired mutation). The resulting cells were karyotypically normal, maintained pluripotency and were able to differentiate into dopaminergic neurons [112]. This study represents one of the first reports of patient-derived iPSCs and wild-type human ESCs being gene edited to create isogenic pairs for disease modelling. This not only represents a significant progress for in vitro disease modelling but also a major advancement towards human iPSC-based cell replacement therapies.

In the context of in vivo applications in the retina, an RNP approach may not be the most suitable, as achieving efficient delivery of the RNP complex into retinal cells may prove difficult. Given the proven efficacy of viral vector-mediated gene transfer to the retina, a gene-based approach, where the Cas9 tool kit is packaged in an AAV vector, may be easier. However, AAV vectors can only package approximately ∼5 Kb of insert and the *Streptococcus pyogenes* Cas9 (SpCas9) DNA is about 4.2 Kb, leaving little or no room for a reporter or gRNA and repair template. Different approaches have been taken to overcome this problem in other cell types. Zhang and colleagues used a dual AAV system approach to deliver SpCas9 and gRNAs to disrupt gene function in dividing mouse brain cells in vivo [113]. The same group later demonstrated that using the SpCas9 orthologue, *Staphylococcus aureus* Cas9 (SaCas9), which is 3.2 Kb and therefore approximately 1 Kb smaller than SpCas9 can be packaged into a single AAV system along with gRNAs and can introduce high levels of gene disruption in mouse liver cells in vivo [114]. Similarly, SaCas9 has been used in both a dual and a single AAV system, with various gRNAs, to target mouse muscle cells in vivo [115]. Recently, Hewitt and colleagues attempted a dual AAV2 CRISPR/Cas9 system approach for disrupting the expression of yellow fluorescent protein (YFP) in retinal ganglion cells of a Thy1-YFP transgenic mouse model [116]. They managed to reduce the number of YFP-positive cells by 84%, apparently without affecting normal retinal function. This proof-of-concept study for NHEJ-based gene editing in the retina, together with the in vivo AAV-based gene editing studies in other tissues, suggests that in vivo retinal gene editing may be achievable at efficiencies high enough for in vivo retinal therapy in the future (Fig. 1). While using AAV to deliver the necessary CRISPR components for efficient gene editing is challenging due to size constraints, other viral vectors are able to accommodate larger DNA inserts. Lentiviral vectors, for example, can package approximately 9 kb of insert DNA that would be large enough to accommodate both a large Cas9 nuclease (e.g. SpCas9) and the gRNA cassettes. One such approach has been used to carry out gene editing in RPE cells where *VEGF-A* was disrupted in vitro in human cells [117]. A similar approach could be used in vivo for targeted gene disruption in the RPE but not for targeting photoreceptor as lentiviral vectors do not transduce these cells efficiently. However, a major problem that still remains to be addressed is how to achieve efficient and accurate HDR-based gene editing in the retina if most of its cells are post-mitotic and largely lack HDR repair.

Together, these studies suggest that disease modelling of a variety of inherited retinal diseases using iPSCs is a realistic prospect. However, the appropriate choice of control cell line and gene editing tool kits to detect subtle phenotypic changes in affected cell types will be critical for successful disease modelling. Regarding in vivo gene editing in the retina, packaging the whole CRISPR/Cas9 gene editing tool kit in a single AAV vector for delivery will likely lead to the highest levels of transduction efficiency and gene disruption in the retina. However, an efficient method to carry out precise DNA substitutions in post-mitotic retinal cells remains to be elucidated.

## Summary and Future Challenges

hPSCs represent a powerful tool for understanding human retinal development, retinal disease modelling, drug discovery and for developing cell transplantation therapies. However, to achieve these successfully, it is necessary to consider a number of requirements. A significant amount of further work is required to establish robust and reproducible protocols for the efficient generation of retinal organoids from hPSC sources. Of particular importance is the ability to generate fully mature hPSC-derived photoreceptors including OS in vitro. This may necessitate the use of bioreactors, co-cultures of RPE cells and/or the use of bioscaffolds. Faithful disease modelling will benefit from the use of isogenic pairs of iPSC lines, as well as robust photoreceptor quantification methods and improved retinal organoid differentiation. Reducing the variability between differentiations remains a major challenge. Generating isogenic controls for the affected cell type should significantly reduce the inherent variability that is introduced by differences in epigenetic and genetic background when using iPSCs from different sources. Methods to assess the phenotype should be quantitative and robust. Patient-specific iPSCs are not only a powerful tool for disease modelling for inherited retinal degeneration but may in the future also become a source of cells for photoreceptor transplantation. Two key aspects we need to consider for cell transplantation studies are the optimal developmental stage of the iPSC-derived retinal tissue to transplant [35, 41, 118], and the genetic correction of the patient’s affected cell type before delivery. Recently, a CD surface marker panel was described for murine ESC-derived photoreceptors. This panel allowed for the isolation and purification of rod photoreceptor cells at the optimal stage of development for transplantation [40, 61]. Promising preliminary studies in human retinal tissue further indicated that a similar panel should be identifiable for hPSC-derived retinal tissue [61], opening the way to isolating photoreceptors for cell transplantation therapies in humans. If iPSCs from patients are to be used as the donor source, it will also be necessary to repair the inherited defect prior to transplantation. CRISPR/Cas9 gene editing is likely to play a key role here. For clinical use, a RNP CRISPR Cas9n gene editing approach is likely to be most advantageous, as it offers high gene editing efficiency and minimum off-targeting effects. A stringent screening of the resulting iPS-derived photoreceptors will be required prior to transplantation; karyotyping, tumorigenicity and whole genome sequencing screens will be necessary to ensure safety in phase I clinical trials. Takahashi and colleagues initiated the first human RPE iPSC-derived phase I clinical trial for neovascular AMD in 2014. The first subject in the trial received autologous iPSC-derived RPE, while subsequent subjects received allogenic iPSC-derived RPE. While several studies have recently shown that hESC-derived RPE cells are safe following transplantation into patients in phase I clinical trials [119–121], some concerns were raised in the study by Takahashi and colleagues about the stability of the genome of the iPSC-derived RPE cells used for sheet transplantation, causing the trial to be temporarily halted. Regardless, this landmark study served as proof-of-principle to show that iPSC-derived cell therapy in humans is possible.

The major advantage of patient-specific, gene-corrected, autologous iPSC-derived cells over hESC-derived cells is reduced immunogenicity following transplantation. However, developing clinical-grade autologous iPSC-derived cells for each individual is likely to be prohibitively expensive for the foreseeable future and alternative strategies are therefore required. In an effort to address this issue, Takahashi and colleagues have recently shown that non-human primate iPSC-derived RPE, transplanted into allogenic major histocompatibility complex (MHC)-matched animals, survived without evidence of rejection while iPSC-derived RPE transplanted into MHC-mismatched animals resulted in immune rejection [122••]. Furthermore, Shinja Yamanaka, Nobel Prize winner for his discovery on iPSCs, is developing a bank of clinical grade iPSC lines that are homozygous for the common Japanese MHC types. This should allow MHC-matched patients to be treated with the same iPSC source, greatly reducing costs and variability of differentiation.

HDR-mediated gene editing in vivo may have potential in the future to treat inherited retinal degenerations, particularly those caused by dominant mutations. Successful in vivo gene disruption via NHEJ using CRISPR/Cas9 in an AAV system has been shown in several tissues. However, HDR-mediated gene editing for precise DNA modifications in post-mitotic neurons remains to be achieved. If HDR-mediated gene editing in post-mitotic retinal cells is achieved using a single AAV system, another aspect to consider will be the requirement to use an inducible CRISPR/Cas9 system where induction of gene editing can be stimulated with a drug and consequently switched off after correction, since long-term expression of Cas9 system could lead to an increase off-targeting of the genome.

Regardless of the undoubted ahead challenges, these are exciting times and the landmark studies discussed in this review open the door to widespread application of hPSC-based technology and gene editing to advance our understanding and treatment of retinal disorders.
